# Insurance acceptance and cash pay rates for psychotherapy in the US

**DOI:** 10.1093/haschl/qxae110

**Published:** 2024-09-09

**Authors:** Jane M Zhu, Aine Huntington, Simon Haeder, Courtney Wolk, K John McConnell

**Affiliations:** Division of General Internal Medicine, Oregon Health and Science University, Portland, OR 97239, USA; Center for Health Systems Effectiveness, Oregon Health and Science University, Portland, OR 97239, USA; Department of Health Policy and Management, Texas A&M University School of Public Health, College Station, TX 77843, USA; Penn Center for Mental Health, Perelman School of Medicine, University of Pennsylvania, Philadelphia, PA 19104, USA; Center for Health Systems Effectiveness, Oregon Health and Science University, Portland, OR 97239, USA

**Keywords:** mental health, psychotherapy, counseling, access to care, cost, self-pay, private practice, insurance

## Abstract

Cost and insurance coverage remain important barriers to mental health care, including psychotherapy and mental health counseling services (“psychotherapy”). While data are scant, psychotherapy services are often delivered in private practice settings, where providers frequently do not take insurance and instead rely on direct pay. In this cross-sectional analysis, we use a large national online directory of 175 083 psychotherapy providers to describe characteristics of private practice psychotherapy providers who accept and do not accept insurance, and assess self-reported private pay rates. Overall, about one-third of private practice psychotherapists did not accept insurance, with insurance acceptance varying substantially across states. We also found significant session rate differentials, with Medicaid rates being on average 40% lower than reported cash pay rates, which averaged $143.26 a session. Taken together, low insurance acceptance across a broad swath of mental health provider types means that access to care is disproportionately reliant on patients’ ability to afford out-of-pocket payments—even when covered by insurance. While our findings are descriptive and may not be representative of all US psychotherapists, they add to scant existing knowledge about the cash pay market for an important mental health service that has experienced increased use and demand over time.

## Introduction

Psychotherapy and mental health counseling services (“psychotherapy”) constitute an important part of outpatient mental health services, both as an adjunct to medication and as a standalone treatment.^1^ Certain types of therapy, including cognitive behavioral therapies, are also considered part of evidence-based treatment for a number of physical health conditions.^2^ As with psychopharmacology services, demand for psychotherapy increased in the wake of the COVID-19 pandemic, driven in part by rising depression, anxiety, and stress-related disorders, particularly in younger populations.^3–5^ At the same time, structural changes in the delivery of mental health services have lowered historical barriers and stigma associated with mental health treatment. For example, statutes like the Mental Health Parity Act of 1996, the Mental Health Parity and Addiction Equity Act of 2008, and the Affordable Care Act's parity requirements have extended insurance coverage to mental health services. Moreover, telehealth use in mental health care has remained high through the pandemic,^6,7^ and nearly all mental health treatment facilities now offer telehealth services.^8^

However, access gaps persist. In 2021, less than half of adults with a mental health condition received any mental health services,^9^ and unmet need among children and adolescents is a persistent concern.^5,10,11^ Cost and insurance coverage remain important barriers to mental health services generally, but may be even more relevant to psychotherapy for several reasons. First, while there is increasing coverage for psychotherapy, mental health provider shortages, high demand have resulted in long wait times, and an increased likelihood of seeking out-of-network care, which is associated with higher out-of-pocket costs.^12–14^ Second, psychotherapy often requires recurrent visits over a prolonged period, which can further exacerbate cost concerns and inequitable access. Finally, while data are scant, psychotherapy services are often delivered in private practice settings, where providers may not take insurance and instead rely on cash pay. One trade group estimate suggests that about 45% of licensed psychologists work in private practice.^15^ In the absence of empirical evidence, lay estimates in the media suggest that between 30% and 50% of these providers may not accept any form of insurance.^16,17^

A large share of recent literature on insurance acceptance has focused on psychiatrists.^18–21^ Less evidence exists for other segments of the mental health workforce, including psychologists, professional counselors, clinical social workers, and others licensed to practice psychotherapy. To fill this gap, we use a large novel dataset to describe the characteristics of those private practice psychotherapy providers who accept insurance and, crucially, those who do not. Because provider participation in some public insurance programs, like Medicaid, may be affected by market factors, including the private cash-pay market (hereafter referred to as cash pay), we also assessed cash pay rates compared to Medicaid rates across states. As administrative claims data do not contain information about cash pay rates, this analysis adds to limited existing knowledge about the private practice market for this important component of mental health care.

## Methods

### Data

Data for this study were collected through web-scraping of an application programming interface (API), a method that allows for automated retrieval of large datasets from online sources. Web-scraping involves programmatically extracting specific data elements from web pages or APIs, which are then structured into a format suitable for analysis. This approach is increasingly used in health services research to gather real-time data from publicly accessible online platforms, including social media, health information websites, and electronic health records systems.^22^ From September 2023 to December 2023, we web-scraped a total of 1.26 million provider listings for psychotherapy and counseling services from *Psychology Today*.^23^*Psychology Today’s* Therapy Directory is an online repository of mental health providers and treatment facilities and is the largest consumer-facing online directory of its kind.^24^ Mental health professionals pay a fixed monthly fee ($29.95) to participate in the directory, and listings are maintained and updated directly by members. Directory information includes addresses, contact information, degrees, licensing, specialty areas of treatment, insurance acceptance, and cash-pay session rates. Data were cleaned to remove duplicate, erroneous, or inactive listings. When data inconsistencies arose, provider listings were manually validated and checked against other web-based sources, including practice websites. *Psychology Today* has a separate database for psychiatrists, which we did not include in this analysis.

Directory listings in *Psychology Today* could either be organizations or individual providers. We identified 11 743 potential group practices in the Psychology Today data, with the remainder being individual psychotherapy and counseling providers. We used probabilistic record linkage algorithms to link exact (street address and zip code) and nonexact records (provider and organization names) to 2023 National Plan and Provider Enumeration System (NPPES) data to further identify individuals affiliated with group practices. A total of 7960 (4.5%) observations were subsequently excluded from our dataset, either because they matched only to organizations in NPPES (*N* = 2864) and we could not ascertain their provider composition or because they were unmatched (*N* = 5096). Our final analytic sample, current as of December 31, 2023, contained 175 083 individual psychotherapy and counseling providers across all 50 states and the District of Columbia (Appendix S1). Of note, due to the nature of the *Psychology Today* database and its membership, this sample (which we call “private practice” providers more broadly) centers on independent solo and small group practitioners who are responsible for financial and business decisions, including rate-setting, and excludes salaried agency psychotherapists in community mental health centers, substance abuse treatment centers, and nonprofit organizations.

### Variables

Our primary outcome was an indicator of any insurance acceptance, a self-reported measure. Providers were grouped into three categories based on highest self-reported titles and degrees: (1) psychologists and other PhDs; (2) Master’s-level therapists and counselors, including licensed clinical social workers, licensed professional counselors, and licensed marriage and family therapists; and (3) all other providers. Binary variables were created for several self-reported provider characteristics: if the provider accepted patients under 18 years of age; if the provider offered telehealth services; and if the provider practiced in a group, estimated by grouping on practice location and/or practice name. Rurality of provider practice location was determined using the 2006 and 2013 NCHS Urban-Rural classification scheme for counties, distinguishing categories 5 (micropolitan) and 6 (noncore).

To mitigate concerns about missingness for reported cash-pay session rates, which could affect the representativeness of our analytic sample, we assessed the extent to which data were missing at random or missing not at random. We visually examined patterns of missingness between missing values and observed values in all variables. We also performed a logistic regression to predict missing session rate data using all previously described variables as predictors. We found no association between the missing indicator and any observed variables.

### Analysis

We used descriptive statistics to assess state- and county-level variation in insurance acceptance, provision of telehealth services, and session rates, and summarized sociodemographic and practice characteristics of those accepting and not accepting insurance.

Due to large sample sizes, we used standardized mean differences to compare means between providers accepting insurance and providers that did not accept insurance. We also compared self-reported session rates to state-based psychotherapy rates in Medicaid fee for service (FFS). Because a standard psychotherapy session is 50–55 min, we compared private pay session rates to Medicaid rates for a 45-min psychotherapy session (Current Procedural Terminology Code 90834). Notably, Medicaid fee schedules in most states do not distinguish differential rates by provider type (eg, billing psychologist vs. psychiatrist). Medicaid reimbursement rates were obtained from publicly available fee schedules and last updated as of October 2022. The methodology for obtaining these rates has been reported elsewhere.^25^ All statistical analyses were performed in R, version 4.3.1.

### Limitations

Our study has important limitations. First, we use a publicly available directory of mental health providers, which is not representative of all private practice psychotherapy providers in the US. Our results thus may not be generalizable to all providers in private practice settings, or to those who practice in academic medical centers, community hospitals, and in government, educational, or business settings. More specifically, our data may exclude psychotherapy providers who are in high demand and do not need to advertise for clients through Psychology Today’s database. Therapists who did not publicly list their cash pay rates also might charge higher rates, which may bias our results. Second, as with any self-reported data, the measures here may be limited by nonresponse bias and subjectivity. Whether or not, for instance, self-reported acceptance of new patients translates to true patient accessibility cannot be verified without secret shopper studies or similar audits. Third, we could not identify acceptance of specific types of insurance, including Medicaid, Medicare, and commercial insurance. We also assessed cash pay rates against Medicaid fee-for-service rates for a comparable service, which may not reflect proprietary negotiated rates under Medicaid managed care. However, studies suggest that fee-for-service rates are often a floor for managed care rates, and overall, managed care rates have been found to be similar and sometimes identical to those paid by Medicaid fee-for-service.^26,27^ Finally, we did not have data on commercial prices, which could also influence insurance participation. Nevertheless, this data represent one of the largest provider directories for mental health providers in the country, has broad geographic coverage, is used exclusively by mental health consumers, and offers a window of visibility into cash pay rates otherwise unavailable in administrative claims data.

## Results

A total of 175 083 psychotherapy and counseling providers were identified. Among these providers, 40.2% (*n* = 70,433) were affiliated with a group practice (Table 1). A large share of providers practiced in the South (33.3%), followed by the West (27.9%), Midwest (20.0%), and Northeast (18.7%); nearly, all providers practiced in metropolitan areas (96.0%). Master’s level counselors and therapists comprised the largest share of providers (76.5%), while psychologists and PhD trained providers comprised 15.6% of providers. The majority of providers (65.1%) reported accepting patients under 18 years of age and a quarter (25.0%) reported offering telehealth.

**Table 1.** Characteristics of psychotherapy and counseling providers, 2023.

	% (N)
	N = 175 083
Part of a group practice	
Yes	40.2 (70 433)
Region	
Midwest	20.0 (35 057)
Northeast	18.7 (32 818)
South	33.3 (58 361)
West	27.9 (48 847)
Rural Classification	
Metro	96.0 (168 073)
Micro	3.0 (5325)
Rural	1.0 (1685)
Provider category	
Psychologists and PhDs	15.6 (27 385)
Master’s level providers	76.5 (133 902)
All other providers	7.9 (13 796)
Accepts insurance	
Yes	64.9 (113 661)
Offers telehealth	
Yes	25.0 (43 745)
Sees patients under 18 years of age	
Yes	65.1 (114 045)
Individual out-of-pocket session cost (USD)	
Mean (SD)	146.76 (46.00)
* *Missing	36.8 (64 346)

Abbreviations: NCHS, National Center for Health Statistics (NCHS); PhD, Doctor of Philosophy or doctoral equivalent; SD, standard deviation; USD, United States dollar.

From authors’ analysis of online provider directory data and NCHS data. Group practices estimated by location name or address. Rural, metro, and microstatus based on provider’s primary practice address, categorized according to the 2013 and 2006 NCHS Urban–Rural Classification Scheme for Counties.

Overall, nearly two-thirds (64.9%) of these providers accepted any insurance (Table 2). Providers who were affiliated with group practices (42.2% vs. 36.6%, SMD = 0.113) were more likely than their counterparts to accept any insurance. Master’s level counselors were more likely to accept insurance compared to psychologists (SMD = 0.148). Providers who accepted insurance also reported lower session costs ($141.06 vs. $155.90) compared to those who did not accept insurance (SMD = 0.315).

**Table 2.** Comparison of psychotherapy and counseling providers, by insurance acceptance.

Accepts insurance	No	Yes	SMD
	(N = 61 422)	(N = 113 661)	
	% (N)	% (N)	
Part of a group practice			0.113
Yes	36.6 (22 508)	42.2 (47 925)	
Rural classification			0.077
Metro	97.0 (59 551)	95.5 (108 522)	
Micro	2.3 (1428)	3.4 (3897)	
Rural	0.7 (443)	1.1 (1242)	
Provider category			0.148
Psychologists and PhDs	19.1 (11 706)	13.8 (15 679)	
Master’s level providers	74.0 (45 478)	77.8 (88 424)	
All other providers	6.9 (4238)	8.4 (9558)	
Offers telehealth			0.036
Yes	24.0 (14 732)	25.5 (29 013)	
Accepts patients under 18 years of age			0.079
Yes	62.7 (38 512)	66.5 (75 533)	
Individual session private pay cost (USD)			0.315
Mean (SD)	155.90 (53.56)	141.06 (39.52)	

Abbreviations: NCHS, National Center for Health Statistics (NCHS); PhD, Doctor of Philosophy or doctoral equivalent; SD, standard deviation; SMD, standardized mean difference; USD = United States dollar.

From authors’ analysis of online provider directory data and NCHS data. Absolute values of >0.1 are suggestive of significant differences. Group practices estimated by location name or address. Rural, metro, and micro status based on provider’s primary practice address, categorized according to the 2013 and 2006 NCHS Urban-Rural Classification Scheme for Counties.

**Figure 1.**
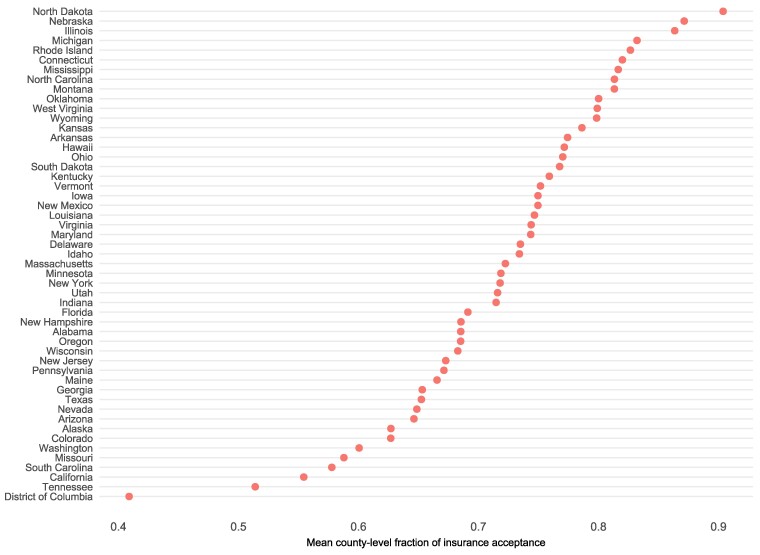
County-level mean fraction of insurance acceptance among private practice psychotherapy and counseling providers, by state. From authors’ analysis of online provider directory data and 5-year ACS data. The X-axis shows the mean county-level fraction of any insurance acceptance among providers. County location was derived from provider’s primary practice address. Insurance acceptance was self-reported.

Insurance acceptance among private practice psychotherapists and counselors varied substantially across states (Figure 1). States with the highest rates of insurance acceptance included North Dakota (90.5%, SD 10.8%), Nebraska (87.1%, SD 22.2%), and Illinois (86.3%, SD 22.0%). In comparison, the District of Columbia (considered a county equivalent) had a mean insurance acceptance rate of 41.0%, followed by Tennessee (51.5%, SD 35.3%) and California (55.4%, SD 24.6%). Session rates differed significantly by insurance status for each provider type (Appendix S2). For example, among psychologists and other PhD-level counselors and therapists, mean session rates for those accepting insurance was $167.69 (SD $48.52), compared to $195.91 (SD $61.52) among those not accepting insurance (*P* < .001). Rate differentials among other provider groups were less pronounced.

**Figure 2.**
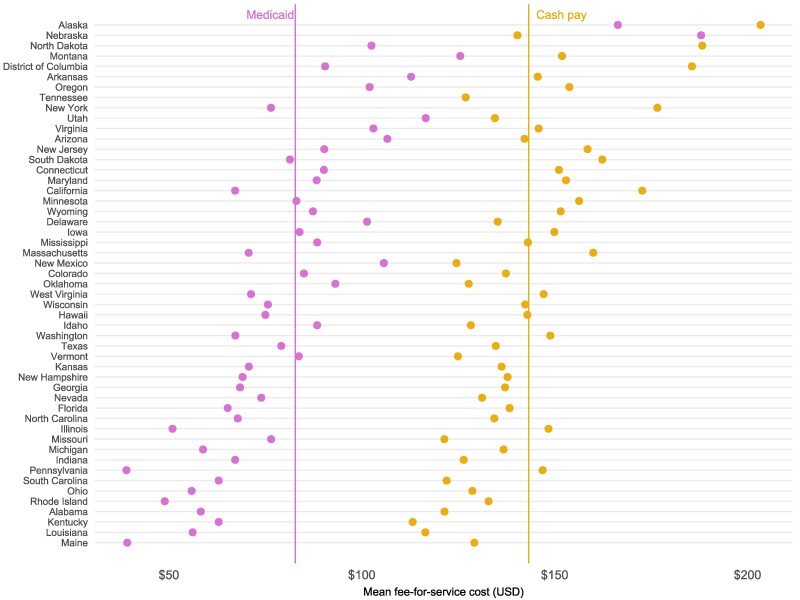
Cash pay session rates among psychotherapy and counseling providers, compared to Medicaid fee-for-service rates, by state. From authors’ analysis of online provider directory data and publicly available Medicaid fee-for-service fee schedules. Medicaid rates shown here are based on a 45 min psychotherapy session (CPT 90834) as of October 2022. Of note, in many states there is no distinction in the fee schedule between different provider types. Medicaid fee-for-service rates for Florida and Mississippi were newly collected using 2022 historical rates. Tennessee does not publish fee schedules and was excluded from calculations of mean differential rates.

Finally, cash pay rates were near-universally higher than Medicaid fee-for-service rates across states (Figure 2). Cash pay rates for a typical session averaged $143.26, compared to $82.77 in Medicaid. Excluding Tennessee, which does not publish Medicaid fee schedules, the mean difference between cash pay and Medicaid rates was $60.81, or -42.4%. The largest rate differentials between cash pay and Medicaid rates were in Pennsylvania ($146.88 vs. $39.00, a difference of 73.4%), California ($172.71 vs. $67.16, a difference of 61.1%), and New York ($176.61 vs. $76.45, a difference of 56.6%). In only one state, Nebraska, was the Medicaid FFS reimbursement rate ($187.96) higher than the reported mean cash pay session rates ($140.34).

## Discussion

In this cross-sectional analysis, we found that, on average, about one-third of private practice psychotherapists did not accept insurance, operating solely in a private cash-pay market. Insurance acceptance also varied substantially across states. Our results, while offering new data on a broad swath of non-physician mental health providers, are largely consistent with other studies that have focused on psychiatrists’ acceptance of insurance. Studies estimate that between 25% and 45% of psychiatrists do not accept any insurance,^18,28^ a higher share than in other physician specialties. The share of psychiatrists accepting public insurance is estimated to be even lower, with the proportion of psychiatrists accepting Medicaid insurance decreasing from 48% to 35% between 2011 and 2015.^19^ Taken together, low insurance acceptance contributes to narrow mental health provider networks across health insurance markets and geographies,^29,30^ and even among those with health insurance coverage, contributes to out-of-network care,^12,31,32^ increased cost-sharing,^14^ and delays in care.^32,33^

It has been posited that low rates of insurance acceptance among mental health providers, particularly within public insurance programs like Medicaid,^34,35^ is driven at least partially by low reimbursement rates.^36–40^ A 2015 American Psychological Association survey of psychologists found that low reimbursement was the primary deterrent for this provider group to accept insurance.^41^ To this end, we found evidence of significant rate differentials between providers accepting insurance and those not accepting any insurance, with particularly notable rate differentials compared to Medicaid fee-for-service. On average, Medicaid rates were ∼40% lower, and as much as 73% lower, than cash pay rates, suggesting a significant financial opportunity cost for providers to accept Medicaid insurance. Our estimates, given data limitations, likely underestimate true cash-pay prices, which signals an even higher gap. Given the outsized role that Medicaid plays in covering individuals with mental health conditions, these rate disparities may serve as a built-in disincentive for providers to treat certain populations despite their disproportionate need. Moreover, a documented shortage of mental health providers, compounded by geographic maldistribution, affords providers the market power to stay outside of insurance networks and to command higher prices in the face of increased service demand. While we were unable to examine rate differentials compared to commercial rates, others have found widening gaps in-network and out-of-network costs, both in terms of prices paid by insurers and cost-sharing for patients.^13^ Thus, private pay rates continue to be higher than what insurance participation could otherwise afford.

Prior research has shown that patients, particularly women and younger age groups,^42^ have demonstrated a consistent preference for psychotherapy over pharmacologic treatment for common mental health concerns, including depression and anxiety.^43^ This may explain, in part, why, despite increasing out-of-pocket costs, utilization of psychotherapy has increased, with higher numbers of visits over time among those with any psychotherapy use.^13^ Nicole Benson and Zirui Song found that in 2017, commercially insured adults receiving in-network psychotherapy had an average 9.4 visits in a single year, while children receiving psychotherapy had 8.4 visits.^13^ A recent study similarly found that one in five outpatient mental health specialist visits for children and adolescents was self-pay, with psychologists and social workers most likely to see this population on a self-pay basis.^44^ Given that cash pay rates averaged above $150 per session in our analysis, these services may only be affordable to limited sectors of the population, exacerbating inequities in access to mental health care that tends to affect lower-income and racial and ethnic minoritized communities disproportionately.^45,46^

Recognizing this burden, state, and federal policymakers have implemented efforts to encourage greater mental health provider participation in insurance networks, particularly public insurance programs like Medicaid and Medicare.^47^ For example, a majority of Medicaid programs now offer individual and group therapy as a covered service, although there remains variation in required copayments and service limits across states.^48^ A number of states, including Oregon, North Carolina, and Virginia, have also engaged in large-scale efforts to improve workforce recruitment and retention, including through reimbursement increases across behavioral health provider types.^49^ The centers for medicare and medicaid services has also introduced payment increases for psychotherapy and counseling services in the outpatient setting in order to increase insurance acceptance in Medicare.^50^ While it is yet unclear whether these efforts will yield new provider participation in insurance markets, or will sustain providers already serving insured populations, these ongoing policies should be closely monitored and evaluated, with specific attention to their effects on access to the psychotherapy workforce.

## Conclusion

Using 2023 online provider directory data, we find that about one-third of private practice psychotherapists and counselors accepted no insurance, and that insurance acceptance varied substantially across states. We also find significant reimbursement rate differentials, with Medicaid rates 40% lower than reported private pay rates. Taken together, low insurance acceptance across a broad swath of mental health provider types means that access to care is disproportionately dependent on patients’ ability to afford out-of-pocket payments—even when covered by insurance. While our findings are descriptive and rely on a database that may not represent all US private practice psychotherapists, they add to scant existing knowledge about private pay rates for an important mental health service that has experienced increased use and demand over time.

## Supplementary Material

qxae110_Supplementary_Data
