# Supplementary material for: Insurance acceptance and cash pay rates for psychotherapy in the US
Source: Health Aff Sch. 2024 Sep 9;2(9):qxae110. doi: 10.1093/haschl/qxae110 (PMC11412241; doi:10.1093/haschl/qxae110)
Supplement: qxae110_Supplementary_Data [file qxae110_supplementary_data.zip › 20240605_Appendix.docx]

*Health Affairs*

**Supplemental Appendix:** **Insurance Acceptance and Cash Pay Rates for Psychotherapy in the U.S.**

Appendix Exhibit 1. Provider counts from Psychology Today directory, by state, 2023

Appendix Exhibit 2. Distribution of session rates, by provider type and insurance acceptance

**Appendix Exhibit 1. Provider counts from Psychology Today directory by state, 2023**

| State | Psychotherapy providers listed on *Psychology Today* |
| --- | --- |
|  | % (N) |
| Alabama | 0.9 (1,550) |
| Alaska | 0.2 (348) |
| Arizona | 2.1 (3,720) |
| Arkansas | 0.7 (1,181) |
| California | 11.0 (19,192) |
| Colorado | 4.5 (7,937) |
| Connecticut | 2.6 (4,580) |
| Delaware | 0.3 (569) |
| District of Columbia | 0.8 (1,377) |
| Florida | 5.0 (8,756) |
| Georgia | 3.5 (6,199) |
| Hawaii | 0.4 (773) |
| Idaho | 0.6 (1,082) |
| Illinois | 5.4 (9,513) |
| Indiana | 1.3 (2,246) |
| Iowa | 0.6 (1,046) |
| Kansas | 0.8 (1,461) |
| Kentucky | 0.9 (1,553) |
| Louisiana | 1.1 (1,846) |
| Maine | 0.5 (890) |
| Maryland | 3.0 (5,225) |
| Massachusetts | 3.9 (6,847) |
| Michigan | 4.0 (6,973) |
| Minnesota | 1.8 (3,099) |
| Mississippi | 0.3 (603) |
| Missouri | 1.5 (2,711) |
| Montana | 0.5 (924) |
| Nebraska | 0.6 (1,114) |
| Nevada | 0.9 (1,557) |
| New Hampshire | 0.5 (797) |
| New Jersey | 4.7 (8,212) |
| New Mexico | 0.5 (940) |
| New York | 5.4 (9,486) |
| North Carolina | 4.3 (7,500) |
| North Dakota | 0.1 (193) |
| Ohio | 2.6 (4,526) |
| Oklahoma | 0.8 (1,421) |
| Oregon | 1.8 (3,090) |
| Pennsylvania | 0.3 (461) |
| Rhode Island | 0.5 (925) |
| South Carolina | 1.2 (,2016) |
| South Dakota | 0.1 (227) |
| Tennessee | 1.8 (3,238) |
| Texas | 5.1 (9,005) |
| Utah | 1.5 (2,713) |
| Vermont | 0.4 (620) |
| Virginia | 3.5 (6,072) |
| Washington | 3.6 (6,327) |
| West Virginia | 0.1 (250) |
| Wisconsin | 1.1 (1947) |
| Wyoming | 0.1 (245) |
| Total | 100 (175,083) |

**Appendix Exhibit 2. Distribution of session rates, by provider type and insurance acceptance**


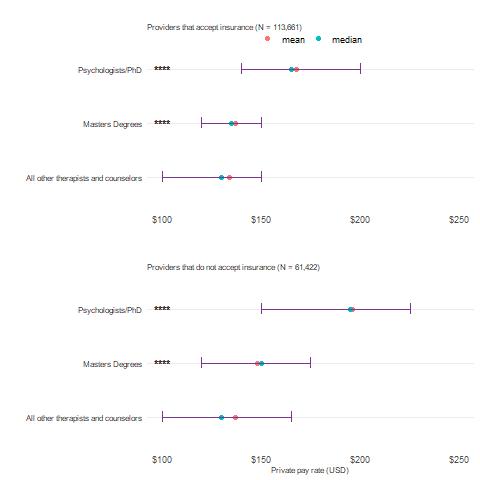


**Notes:** From authors’ analysis of online provider directory data. Exhibit 2 shows mean, median, and interquartile range (IQR, 25^th^-75^th^ percentile) of session rates by provider type, for those providers accepting any insurance vs. accepting no insurance. A star denotes statistical significance (p<0.001) for between-group comparisons (same provider type, across insurance status) using a two-way ANVOA with Tukey’s HSD post-hoc.
